# Routine immunisation programmes in southeast Asia: beyond the routine

**DOI:** 10.1016/j.lansea.2022.100138

**Published:** 2023-01-05

**Authors:** The Lancet Regional Health – Southeast Asia

Routine immunisation is a cost-effective strategy for the control of vaccine-preventable diseases. The COVID-19 pandemic and associated lockdowns has affected routine immunisation programmes globally; and recent disease outbreaks in the WHO South-East Asia Region (SEAR) show that more actions are needed.

The WHO SEAR was declared polio-free in 2014. However, in November, 2022, four new cases of polio were reported in the Aceh province of Indonesia. In 2017, the Indonesian Government vaccinated children against rubella and measles while omitting the polio vaccine. The Indonesian Government now plans to provide polio vaccinations for the 1.2 million children in Aceh province. In Bangladesh, where cholera is endemic due to regular floods, annual cholera vaccination programmes are in place. However, as a result of climate change, the risk of cholera outbreaks in Bangladesh is predicted to increase, thus vaccinations should be prioritised for people living at low land elevation and near surface water.

In India, a retrospective study by Summan and colleagues of the National Family Health Survey (2019–21) data showed that routine immunisation coverage had decreased among children due to the COVID-19 pandemic and subsequent delays to vaccination programmes. As of December 8, 2022, a measles outbreak in India, which has been especially severe in the state of Maharashtra, has resulted in 17 deaths, which highlights the effect of lower routine immunisation coverage in various parts of the country. It is well known that the risk of measles is higher among individuals who are immunocompromised, vitamin-deficient, and malnourished, and risk of mortality is associated with the individual's health and socioeconomic status. Only after the outbreak did health authorities prioritise malnourished children for measles vaccination and vitamin A supplementation, which is of substantial concern. Furthermore, health authorities had no available data on the number of children who missed the second dose of vaccine, which could be attributed to the COVID-19 pandemic. In Pakistan, 39 children died from diphtheria due to unavailability of vaccines. The WHO and the UNICEF have currently promised to supply anti-diphtheria sera to Pakistan.

Immunisations are important because they contribute to the achievement of herd immunity. For example, a high seropositivity in the adult population against SARS-CoV-2 had indirectly provided protection to unvaccinated children in India. Several states in India opted to reopen schools after the delta (B.1.617.2) variant wave. A study by Mozaffer and colleagues used mathematical modelling to show that reopening of schools in India would not lead to a substantial increase in COVID-19 cases. Hybrid immunity (acquired by natural infection and vaccination) in the population and decreased severity of the omicron (B.1.1.529) variant led to fewer cases and hospital admissions than delta wave. However, vaccine hesitancy remains an issue. Adverse events associated with vaccination can contribute to hesitancy and could be addressed by a preliminary diagnosis. For example, seizures associated with diphtheria, tetanus, and whole-cell pertussis (DTwP) vaccination is one of the main reasons for vaccine hesitancy in India. A cross-sectional study by Negi and colleagues showed that genetic testing using next-generation sequencing is important for the diagnosis of DTwP vaccination-associated seizures. The authors identified 33 pathogenic variants of 12 genes among 54 Indian children with DTwP vaccination-associated seizures. Since pathogenic variants were mostly identified in the *SCN1A* gene, this proof-of-concept study suggests that Sanger sequencing of only the *SCN1A* gene as a preliminary diagnosis could be a solution. However, further research on more cost-effective diagnostic methods is required. Another approach would be to switch to diphtheria, tetanus, and acellular pertussis (DTaP) because the acellular pertussis component results in fewer adverse effects, however, the DTaP vaccine is more expensive.

During the process of routine immunisation, disease burden in the population becomes lower, and parents might subsequently perceive that immunising their children is not important. Additionally, governments might reduce the budget for the immunisation programmes to focus on other diseases or investments. Hence, the efforts of governments within SEAR to increase public awareness and mitigate the dissemination of false information with the aim of maintaining high levels of vaccination coverage should be continued. Almost a quarter of the global population reside in SEAR, thus health authorities should refrain from considering outbreaks as isolated cases and using other metrics such as cases per unit population to lessen the perceived severity of such outbreaks. Health authorities should not become complacent when comparing the success of vaccination programmes with those of other countries, and instead encourage collaboration among countries. Only by adhering to routine immunisation programmes and ensuring high vaccine coverage can the lives of our communities be protected.

